# Diabetes State Atlas Now Available Online

**Published:** 2014-11-21

CDC’s Division of Diabetes Translation announces the launch of the Diabetes State Atlas (available at http://www.cdc.gov/diabetes/data), an interactive Internet tool for the public to view maps and charts of diabetes data and trends at the U.S. state level. Some of the features of the atlas include 1) customizable maps and graphics of diabetes surveillance data, 2) an interactive application to view state-specific trends by age and sex, and 3) downloadable maps, charts, and data tables that can be used in grant applications, reports, articles, and publications.

The Diabetes State Atlas can help state public health officials document the burden of diabetes in their states, monitor trends, identify high-risk groups and assess disparities between groups, and track progress in achieving *Healthy People 2020* diabetes objectives (1).

In the United States, about 29 million persons have diabetes (2). An additional 86 million adults have prediabetes, putting them at increased risk for developing type 2 diabetes, heart disease, and stroke (2). However, persons with diabetes can take steps to control the disease and prevent complications, and those with prediabetes can prevent or delay the onset of type 2 diabetes through weight loss and physical activity (3). Information about diabetes prevention and control is available online from CDC’s Division of Diabetes Translation at http://www.cdc.gov/diabetes/home/index.html.
